# Association between enterocyte injury and fluid balance in patients with septic shock: a post hoc exploratory analysis of a prospective observational study

**DOI:** 10.1186/s12871-021-01515-2

**Published:** 2021-11-23

**Authors:** Haruka Yokoyama, Motohiro Sekino, Hiroyuki Funaoka, Shuntaro Sato, Hiroshi Araki, Takashi Egashira, Rintaro Yano, Sojiro Matsumoto, Taiga Ichinomiya, Ushio Higashijima, Tetsuya Hara

**Affiliations:** 1 Department of Anesthesiology and Intensive Care Medicine, Nagasaki University Graduate School of Biomedical Sciences, 1-7-1 Sakamoto, Nagasaki, 852-8501 Japan; 2 Department of Research and Development, SB Bioscience Co. Ltd., 3-47 Higashi-Tsukaguchi-cho, 2-chome, Amagasaki, Hyogo 661-0011 Japan; 3 Clinical Research Center, Nagasaki University Hospital, 1-7-1 Sakamoto, Nagasaki, 852-8501 Japan; 4 Department of Intensive Care, Nagasaki Harbor Medical Center, 6-39 Shinchi-machi, Nagasaki, 850-8555 Japan

**Keywords:** Enterocyte damage, Fluid administration, Fluid volume, Intensive care unit, Intestinal fatty acid-binding protein, Intestinal mucosal cell, Sepsis, Septic shock

## Abstract

**Background:**

The required fluid volume differs among patients with septic shock. Enterocyte injury caused by shock may increase the need for fluid by triggering a systematic inflammatory response or an ischemia-reperfusion injury in the presence of intestinal ischemia/necrosis. This study aimed to evaluate the association between enterocyte injury and positive fluid balance in patients with septic shock.

**Methods:**

This study was a post hoc exploratory analysis of a prospective observational study that assessed the association between serum intestinal fatty acid-binding protein, a biomarker of enterocyte injury, and mortality in patients with septic shock. Intestinal fatty acid-binding protein levels were recorded on intensive care unit admission, and fluid balance was monitored from intensive care unit admission to Day 7. The association between intestinal fatty acid-binding protein levels at admission and the infusion balance during the early period after intensive care unit admission was evaluated. Multiple linear regression analysis, with adjustments for severity score and renal function, was performed.

**Results:**

Overall, data of 57 patients were analyzed. Logarithmically transformed intestinal fatty acid-binding protein levels were significantly associated with cumulative fluid balance per body weight at 24 and 72 h post-intensive care unit admission both before (Pearson’s *r* = 0.490 [95% confidence interval: 0.263–0.666]; *P* < 0.001 and *r* = 0.479 [95% confidence interval: 0.240–0.664]; *P* < 0.001, respectively) and after (estimate, 14.4 [95% confidence interval: 4.1–24.7]; *P* = 0.007 and estimate, 26.9 [95% confidence interval: 11.0–42.7]; *P* = 0.001, respectively) adjusting for severity score and renal function.

**Conclusions:**

Enterocyte injury was significantly associated with cumulative fluid balance at 24 and 72 h post-intensive care unit admission. Enterocyte injury in patients with septic shock may be related to excessive fluid accumulation during the early period after intensive care unit admission.

## Background

Sepsis is responsible for considerable morbidity and mortality, with most deaths occurring due to cardiovascular or multiorgan failure [1]. Fluid therapy is essential for early resuscitation in patients with septic shock to increase cardiac output and improve organ perfusion; however, optimal targets and fluid therapy strategies are yet to be established [2, 3].

Several studies have demonstrated an association between positive fluid balance and poor outcomes in patients with septic shock [4–7]. Recently, large database studies have also reported an association between prognosis and the amount of fluid infused or fluid balance in the early period of intensive care unit (ICU) admission, especially within 24–72 h [8, 9]. Fluid overload causes multiple organ dysfunction due to edema in the pulmonary, renal, and cardiovascular systems, worsening the prognosis [10]. Therefore, it stands to reason that fluid volume restriction may improve prognosis. However, the effects of such restrictions remain unproven [11]. Infusion of excess fluid that deviates from the appropriate preload should be avoided, and fluid restriction must be applied to improve the prognosis. However, other factors such as enterocyte injury may promote a positive fluid balance due to increased fluid requirements and may also be associated with a poor prognosis.

Enterocytes comprise more than 80% of all intestinal epithelial cells and act as a barrier to translocation of luminal antigens, and microbiota and their toxic products into the circulation [12]. Hypoperfusion injury of the enterocytes destroys this barrier and triggers a systemic inflammatory response, which can lead to multiple organ dysfunction and poor outcomes [13–15]. Enterocyte injury itself may increase the need for fluid by triggering a systemic inflammatory response or ischemia-reperfusion injury when resulting intestinal ischemia or necrosis occurs [16]. In other words, patients with septic shock who present with enterocyte injury may have a more positive fluid balance than those without enterocyte injury. However, to our knowledge, the association between enterocyte injury and positive fluid balance in patients with septic shock has not been evaluated. If an association is identified, it may help individualize strategies to promote an optimal fluid balance in patients with septic shock.

We investigated the relationship between enterocyte injury in patients with septic shock and fluid balance during the early period after ICU admission. We hypothesized that, in patients with septic shock, enterocyte injury at ICU admission is associated with an excessive positive fluid balance 24- and 72-h post-ICU admission.

## Methods

### Study design

This was a post hoc exploratory analysis of data obtained from a prospective observational study that evaluated the association between enterocyte injury and patient outcomes in patients with septic shock [17, 18]. Additional fluid management data were collected, along with the original study’s data, and additional statistical tests were performed to address new clinical questions. The original study was conducted in an eight-bed general ICU at Nagasaki University Hospital, Nagasaki, Japan, between May 2012 and March 2015. Ethical approval for this post hoc analysis was obtained from the Institutional Review Board of Nagasaki University Hospital (approval number: 18111922). The requirement for written informed consent was waived owing to the retrospective nature of the study.

### Study population

A total of 57 patients with septic shock, who required mechanical ventilation, were enrolled in the original study. Septic shock was defined as acute organ dysfunction in the presence of infection with a need for vasopressors [19]. The original study excluded patients with confirmed or strongly suspected small bowel ischemia with or without necrosis at the source of sepsis, a medical history of extensive small bowel resection, chronic small bowel disease, pregnancy, uncontrolled bleeding, and those in the terminal stages of any comorbidity.

### Data collection

The database from the original study and newly collected data were used in this study. The baseline values at ICU admission and the values within the first 24 h of admission were taken from the original study’s database, which included age, sex, body mass index, Acute Physiology and Chronic Health Evaluation (APACHE) II score, Sequential Organ Failure Assessment score, Japanese Association for Acute Care Medicine (JAAM) disseminated intravascular coagulation (DIC) score [20], mean arterial pressure, heart rate (HR), central venous pressure (CVP), inotropic score [21], blood gas analysis data (PaO_2_/F_I_O_2_ ratio, pH, and HCO_3_^−^), ventilator settings (positive end-expiratory pressure and peak inspiratory pressure), and post-admission interventions for septic shock management, including the administration of vasopressin, low-dose steroids, continuous renal replacement therapy, or polymyxin B-direct hemoperfusion. Estimated glomerular filtration rate (eGFR) at ICU admission was calculated using the equation for Japanese patients recommended by the Japanese Society of Nephrology: eGFR (mL/min/1.73 m^2^) = 194 × Cr (mg/dL)^-1.094^ × age^-0.287^ (if female, × 0.739) [22]. Admission route, surgical interventions, site of infection, and bacteremia status were also included. Patient outcomes, including all-cause 28-day mortality and in-hospital mortality, were recorded.

Standard blood chemistry tests were performed at ICU admission to evaluate sepsis severity, including arterial levels of serum lactate, N-terminal pro-B-type natriuretic peptide, and procalcitonin. These data were obtained from records maintained by Nagasaki University Hospital.

We collected retrospective data on fluid management from the ICU information system (Prescient ICU; FUJIFILM Medical Co. Ltd., Tokyo, Japan). Data were collected for the first 7 days of ICU admission. The first day of ICU admission was defined as the time between ICU admission and 24 h. Daily fluid intake was calculated as the sum of all intravenous and oral fluids (including blood products and nutritional supplements). Daily fluid output was calculated as the sum of the urine volume, ultrafiltration volume, drainage volume, and gastrointestinal loss. Daily fluid balance was calculated by subtracting the total fluid output from the total fluid intake. For patients who died or were discharged from the ICU within 7 days, the fluid balance was calculated up to the day before the date of death or discharge.

### Assessment of enterocyte injury

Serum intestinal fatty acid-binding protein (I-FABP) was used as a biomarker of enterocyte injury. Serum I-FABP levels were provided by an external laboratory (DS Pharma Biomedical [now SB Bioscience] Co. Ltd., Osaka, Japan) using a human I-FABP-specific enzyme-linked immunosorbent assay. The mean serum I-FABP level in healthy adult volunteers was 1.1 ± 0.9 ng/mL (range, 0.1–5.5) [23], and the cutoff value for diagnosing small bowel ischemia was 9.1 ng/mL [24]. ICU physicians were blinded to the patients’ I-FABP levels.

### Clinical management

During the ICU stay, the fluid management plan was determined at the discretion of experienced ICU physicians. Fluid management was based on echocardiography, volume responsiveness to blood pressure and HR, and CVP, as appropriate; however, there was no specific, standardized fluid management plan. Blood products were used based on experienced ICU physicians’ orders.

Details regarding the patients’ clinical management have been described previously [17, 18]. All patients were principally treated as per the Surviving Sepsis Campaign guidelines [19], except for Japan-specific treatment options, such as polymyxin B-direct hemoperfusion [25], and the administration of recombinant soluble thrombomodulin and antithrombin for septic DIC [26, 27].

### Statistical analysis

Clinical data are presented as medians and interquartile ranges for quantitative variables and as frequencies and percentages for categorical variables. I-FABP levels and eGFR were logarithmically transformed to normalize their distributions. The correlation between cumulative fluid balance per body weight at 24- and 72-h post-ICU admission and I-FABP levels at ICU admission were evaluated using Pearson’s correlation coefficient (r). A multiple linear regression analysis was performed to evaluate the association between cumulative fluid balance per body weight at 24- and 72-h post-ICU admission and I-FABP levels at ICU admission. The non-renal APACHE II score (APACHE II score without the renal component) and eGFR at ICU admission were adopted as confounders. The APACHE II score was used as the sepsis severity score and was directly suggestive of fluid balance [9]. Furthermore, eGFR was used to assess renal function, as it is associated with I-FABP clearance and affects I-FABP levels [28]. All tests were two-sided, and *P*-values < 0.05 were considered statistically significant. All statistical analyses were performed using JMP Pro v15 (SAS Institute Inc., Cary, NC, USA) and R version 3.5.0 (R Foundation for Statistical Computing, Vienna, Austria).

## Results

### Study population

Fifty-seven patients with septic shock, who required mechanical ventilation, were included in this post hoc analysis. Their baseline characteristics are summarized in Table 1. Most patients were older and had a more severe shock. The proportion of patients diagnosed with DIC using the JAAM DIC score was 72%. Eleven patients had hospital-onset septic shock, while the remaining 46 patients had an out-of-hospital shock. Twenty-two patients required surgery for source control before ICU admission. The most common source of infection was the abdomen (33%), followed by the lung/thorax (21%), soft tissues (16%), urinary tract (7%), and others (23%). Blood cultures were positive in 54% of the patients. The overall 28-day and in-hospital mortality rates were 23% (13/57) and 32% (18/57), respectively.

**Table 1:** Baseline characteristics of patients with septic shock

Characteristics	Median (IQR)
Age, years	71 (62–79)
Male, n (%)	35 (61)
Body mass index, kg/m^2^	22 (19–24)
APACHE II score	30 (25–36)
SOFA score	13 (11–15)
JAAM DIC score	5 (3–7)
Baseline data and setting at ICU admission	
Mean arterial pressure, mmHg	81 (73–90)
Heart rate, beats/min	103 (92–113)
Central venous pressure, mmHg	11 (9–14)
Inotropic score ^a^	40 (25–55)
PaO_2_/F_I_O_2_ ratio	227 (174–290)
PEEP, cmH_2_O	8 (5–8)
Peak inspiratory pressure, cmH_2_O	21 (20–23)
pH	7.29 (7.26–7.34)
HCO_3_^−^, mmol/L	17.7 (15.5–20.2)
Lactate, mmol/L	3.0 (2.1–6.8)
Procalcitonin, ng/mL	42.5 (10.7–118.2)
NT-proBNP, ng/mL	9585 (2388–24,389)
eGFR, mL/min/1.73 m^2 b^	20 (11–38)
Intervention after ICU admission	
Vasopressin, n (%)	28 (49)
Steroid, n (%)	46 (81)
Continuous renal replacement therapy, n (%)	46 (81)
Polymyxin B-direct hemoperfusion, n (%)	31 (54)

*Abbreviations*: *APACHE* Acute Physiology and Chronic Health Evaluation, *DIC* Disseminated intravascular coagulation, *eGFR* Estimated glomerular filtration rate, *ICU* Intensive care unit, *IQR* Interquartile range, *JAAM* Japanese Association for Acute Care Medicine, *NT-proBNP* N-terminal pro-B-type natriuretic peptide, *PEEP* Positive end-expiratory pressure, *SOFA* Sequential Organ Failure Assessment

Data are reported as median (IQR), unless otherwise indicated

^a^ Inotropic score calculated as (dopamine dose × 1) + (dobutamine dose × 1) + (epinephrine dose × 100) + (norepinephrine dose × 100), where all doses are expressed in micrograms per kilogram per minute

^b^ eGFR calculated as 194 × Cr (mg/dL)^-1.094^ × age^-0.287^ (if female, × 0.739)

### Daily fluid balance and I-FABP levels over the first week of ICU admission

**Fig. 1:**
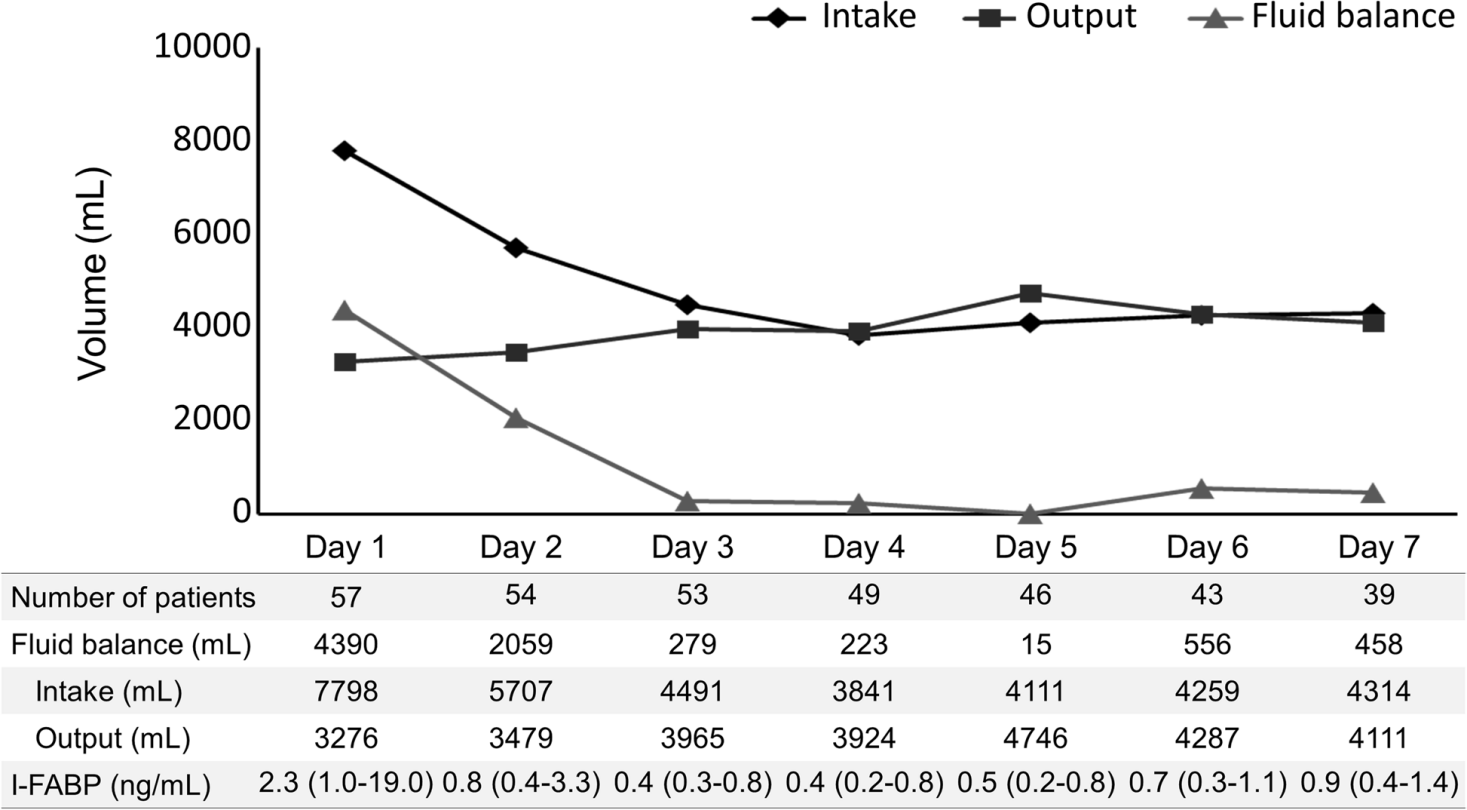
Daily median fluid balance and I-FABP levels during the first 7 days in ICU. Daily median fluid balance, intake, output, and intestinal fatty acid-binding protein (I-FABP) levels over the first 7 days of intensive care unit (ICU) admission. A positive fluid balance was observed during the early post-ICU admission period, especially on ICU Day 1; however, an almost even balance was observed after ICU Day 3. I-FABP levels rapidly decreased after ICU admission. Data are reported as median or median (interquartile range)

Figure 1 illustrates the median daily fluid balance and I-FABP levels over the first week of ICU admission. Positive fluid balances were observed during the early post-ICU admission period, especially on ICU Day 1. However, an almost even balance was observed after ICU Day 3. The median cumulative fluid balance values on ICU Day 1 (24 h after ICU admission) and ICU Day 3 (72 h after ICU admission) were 4390 (2588–6636) mL and 6409 (4018–11,531) mL, respectively. I-FABP levels rapidly decreased after ICU admission.

### Correlation between I-FABP levels at ICU admission and cumulative fluid balance at 24 and 72 hours after ICU admission

**Fig. 2:**
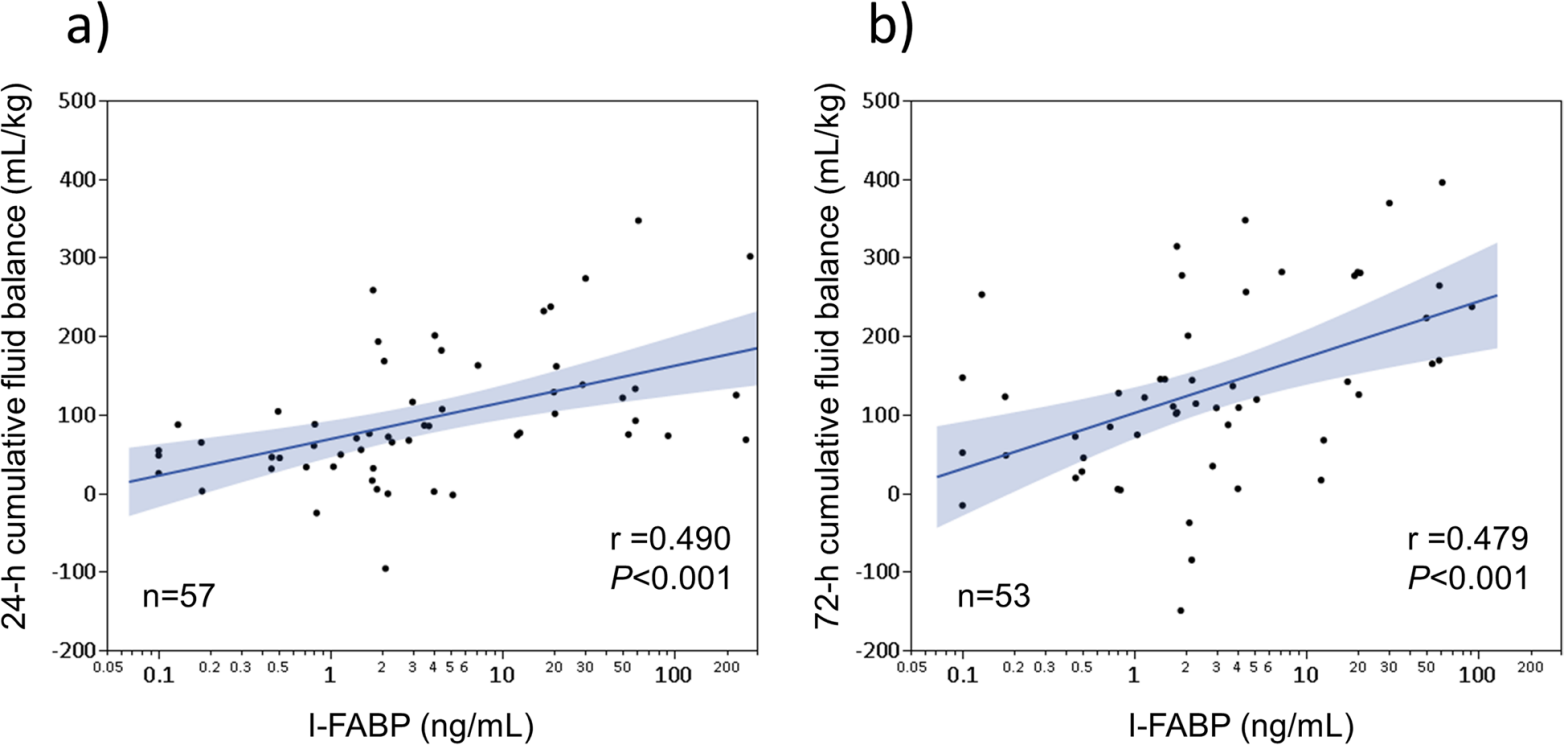
Correlation between I-FABP levels and cumulative fluid balance. Correlation between intestinal fatty acid-binding protein (I-FABP) levels and cumulative fluid balance from intensive care unit (ICU) admission to **(a)** 24 and **(b)** 72 h post-ICU admission. Significant correlations were observed between I-FABP levels and cumulative fluid balance in both analyses (Pearson correlation test)

At ICU admission, I-FABP levels were significantly correlated with the 24- and 72-h cumulative fluid balance (Pearson’s *r* = 0.490 [95% confidence interval (CI): 0.263–0.666]; *P* < 0.001 and *r* = 0.479 [95% CI: 0.240–0.664]; *P* < 0.001, respectively) (Fig. 2a, b).

### Association between fluid balance at 24 and 72 hours and I-FABP levels at ICU admission adjusted for severity score and renal function

The results of multiple linear regression analysis of cumulative fluid balance per body weight at 24- and 72-h post-ICU admission and I-FABP levels at ICU admission, adjusted for non-renal APACHE II score and eGFR, are summarized in Table 2. I-FABP levels were significantly associated with cumulative fluid balance at both 24 (estimate, 14.4 [95% CI: 4.1–24.7]; *P* = 0.007) and 72 h (estimate, 26.9 [95% CI: 11.0–42.7]; *P* = 0.001) post-ICU admission. In other words, a 1% increase in the I-FABP level increases the cumulative infusion balance by 0.144 mL per kg of body weight at 24 h and by 0.269 mL per kg of body weight at 72 h after ICU admission.

**Table 2:** Multiple linear regression analysis of fluid balance and I-FABP adjusted for severity score and eGFR

Dependent variables	Independent variables: log I-FABP			
	Estimate ^a^	95% CI		*P*-value
		Lower limit	Upper limit	
24-h fluid balance/BW	14.4	4.1	24.7	0.007^b^
72-h fluid balance/BW	26.9	11.0	42.7	0.001^b^

*Abbreviation*s: *APACHE* Acute Physiology and Chronic Health Evaluation, *BW* Body weight, *CI* Confidence interval, *eGFR* Estimated glomerular filtration rate, *I-FABP* Intestinal fatty acid-binding protein

^a^ Adjusted for non-renal APACHE II score and log eGFR

^b^ Statistically significant

## Discussion

I-FABP level at ICU admission was significantly associated with cumulative fluid balance at 24- and 72-h post-ICU admission, both before and after adjusting for sepsis severity score and renal function. In patients experiencing septic shock, enterocyte injury may be related to an excessive positive fluid balance during the early period after ICU admission.

Fluid requirements vary widely among patients, and factors associated with increased fluid requirements in patients with septic shock remain unclear. In a recent study, which reported an association between the volume of fluid infused during the first 24 h after ICU admission and prognosis, a large range of fluid volumes were administered during the first 24 h after ICU admission; these volumes ranged from 2000 mL (10th percentile) to 9288 mL (90th percentile) among patients with septic shock who required mechanical ventilation [8]. In addition to more severe illness, older age, postoperative and immunosuppressed status, positive blood cultures, and sepsis with an abdominal source of infection were cited as factors potentially related to a positive fluid balance in the first 24 h after ICU admission [9]. Since intestinal ischemia/necrosis is assumed to be the commonest source of abdominal infection, we believe that this result supports our findings. Enterocyte injury followed by a systemic inflammatory response and possible intestinal ischemia/necrosis may significantly affect the fluid balance during the early period after ICU admission.

To improve the prognosis of patients with septic shock, iatrogenic fluid overload should be avoided. However, uniform fluid restrictions in patients with increased fluid requirements may lead to hypovolemia and a worse prognosis. Studies that evaluated the effectiveness of protocol-based fluid restriction reported that interventions could suppress infusion balance and reduce the incidence of renal injury [29, 30]; however, the effects of such a protocol on prognosis are unknown [11]. Additionally, protocol violations and deviations were reported in the infusion-restricted group [29, 30]. These violations could have resulted from the physicians’ opinions that infusion restrictions did not benefit some of their patients. There is an urgent need to identify factors associated with increased fluid requirements in patients with septic shock to provide individualized fluid management strategies.

Enterocyte injury is reportedly associated with poor clinical outcomes in patients with critical illness [31], acute heart failure, cardiogenic shock [32], postoperative cardiac surgery [33, 34], and septic shock—as demonstrated in our original study [17, 18]. Enterocyte injury increases the fluid requirements and may be associated with a worse prognosis. Our original study revealed that I-FABP levels at ICU admission were associated with 28-day mortality and the incidence of non-occlusive mesenteric ischemia [17]. In patients who present with enterocyte injury, gut-derived sepsis and hidden intestinal ischemia/necrosis should be addressed while optimizing infusion volume to improve prognosis. However, the question of whether enterocyte injury is an independent risk factor associated with increased fluid requirements and poor prognosis should be elucidated in future, well-powered studies.

To our knowledge, this is the first report investigating the association between enterocyte injury at ICU admission and fluid balance during the early period after ICU admission. An excessive positive fluid balance can be caused by patient-related factors, as well as iatrogenic fluid overload. Elucidation of patient-related factors is necessary to determine individual fluid needs and improve the prognosis of patients with septic shock. We believe that this study is crucial because our findings suggest that enterocyte injury may be one such factor with prognostic significance.

This study has several limitations. First, there are inherent limitations to post hoc exploratory analyses, and our patient cohort was relatively small. Therefore, our findings require confirmation through the prospective examination of a larger cohort. Second, we did not have access to information about the patients’ pre-ICU admission fluid volumes and outputs. It was difficult to retrospectively collect accurate data before admission to our hospital, either in the ward or emergency room. At ICU admission, our patients’ mean arterial pressure, HR, and CVP values were within a relatively narrow range; therefore, we presume that there were no significant differences in their respective pre-admission fluid needs. Although differences in fluid balance before ICU admission may have affected our results, other studies have likewise analyzed fluid balance solely after ICU admission [4–9]. Third, the median CVP value on ICU admission in this study was relatively high; therefore, congestion may have caused the enterocyte injury. A study examining the association between venous congestion, assessed by invasively measured hemodynamic parameters, and enterocyte injury in patients with acute decompensated heart failure found no association between them [35]. If enterocyte injury was caused by congestion due to excessive fluid infusion before and after ICU admission, the I-FABP levels should have increased at ICU admission or after ICU admission; however, as shown in Fig. 1, the I-FABP levels decreased after ICU admission, and there was no correlation between CVP at ICU admission and I-FABP levels at ICU admission in this study (Pearson’s *r* = − 0.035; *P* = 0.797). Theoretically, congestion can also cause small enterocyte injury; however, we believe that the primary cause of enterocyte injury in this study was hypoperfusion due to septic shock.

## Conclusions

Our post hoc exploratory analysis found that enterocyte injury at ICU admission was significantly correlated with fluid balance, particularly during the early period—within 24 and 72 h—post-ICU admission, both before and after adjusting for sepsis severity and renal function. In patients with septic shock, enterocyte injury may contribute to excessive fluid accumulation during the early period after ICU admission.

## Data Availability

The datasets used and analyzed during the current study are available from the corresponding author on reasonable request.

## Abbreviations

APACHE: Acute Physiology and Chronic Health Evaluation
CI: Confidence interval
CVP: Central venous pressure
DIC: Disseminated intravascular coagulation
eGFR: Estimated glomerular filtration rate
HR: Heart rate
ICU: Intensive care unit
I-FABP: Intestinal fatty acid-binding protein
JAAM: Japanese Association for Acute Care Medicine
